# The Validity of Online Patient Ratings of Physicians: Analysis of Physician Peer Reviews and Patient Ratings

**DOI:** 10.2196/ijmr.9350

**Published:** 2018-04-09

**Authors:** Robert J McGrath, Jennifer Lewis Priestley, Yiyun Zhou, Patrick J Culligan

**Affiliations:** ^1^ University of New Hampshire Durham, NH United States; ^2^ Graduate College Kennesaw State University Kennesaw, GA United States; ^3^ Department of Urology Weill Cornell Medicine New York, NY United States

**Keywords:** physician review websites, online patient ratings, physician peer review

## Abstract

**Background:**

Information from ratings sites are increasingly informing patient decisions related to health care and the selection of physicians.

**Objective:**

The current study sought to determine the validity of online patient ratings of physicians through comparison with physician peer review.

**Methods:**

We extracted 223,715 reviews of 41,104 physicians from 10 of the largest cities in the United States, including 1142 physicians listed as “America’s Top Doctors” through physician peer review. Differences in mean online patient ratings were tested for physicians who were listed and those who were not.

**Results:**

Overall, no differences were found between the online patient ratings based upon physician peer review status. However, statistical differences were found for four specialties (family medicine, allergists, internal medicine, and pediatrics), with online patient ratings significantly higher for those physicians listed as a peer-reviewed “Top Doctor” versus those who were not.

**Conclusions:**

The results of this large-scale study indicate that while online patient ratings are consistent with physician peer review for four nonsurgical, primarily in-office specializations, patient ratings were not consistent with physician peer review for specializations like anesthesiology. This result indicates that the validity of patient ratings varies by medical specialization.

## Introduction

In a 2016 study, the Pew Research Center found that 84% of all adults in the United States use online ratings sites to inform their product or service purchase decisions [1]. The same is true for health care: patients increasingly access online ratings sites to inform their health care decisions, with online ratings emerging as the most influential factor for choosing a physician. In a 2017 study by the National Institutes of Health, 53% of physicians and 39% of patients reported visiting a health care rating website at least once [2]. Overall, physicians indicated that the numerical results from these ratings websites were valid approximately 53% of the time, while patients indicated that they thought the ratings were valid 36% of the time [2].

RateMDs.com, HealthGrades.com, and Vitals.com are three frequently visited health care provider ratings websites, with over 2.6 million, 6.1 million, and 7.8 million reviews, respectively [3-5]. For these three sites, numeric rating scales range from 1 (poor) to 5 (excellent) and cover perceptions of physician knowledge, helpfulness, punctuality, and staff. Most patients give physicians positive ratings: one study reported that over 90% of all ratings were positive [6] and another reported that as the frequency of ratings increased, the average mean rating increased [7].

Extending the findings of the study by the National Institutes of Health, we sought to determine the validity of online patient ratings through comparison with physician peer review, defined in this study through Castle Connolly Medical. Specifically, we tested whether mean online patient ratings for physicians, by specialty, are higher for those physicians who have been nominated by their peers as one of “America’s Top Doctors” or not, as reported by Castle Connolly Medical. If online patient ratings were consistent with Castle Connolly Medical, ratings for physicians listed would be higher than for those not listed, thereby providing support for the validity of physician online review sites to inform health care-related decisions.

## Methods

The basis for physician peer review selected for the current study is Castle Connolly Medical, a private consumer research firm that distinguishes top providers both nationally and regionally through a peer nomination process that involves over 50,000 providers and hospital and health care executives. Castle Connolly Medical receives over 100,000 nominations each year and a physician-led research team awards top providers from these nominations [8]. Lists are generated for each health care specialty as well as most subspecialties.

Several studies have similarly selected physician peer review through Castle Connolly Medical as a basis to assess the validity and role of patient online ratings sites, including an assessment for hand surgeons in the United States [9], as well as a more general correlation of physician attributes and ranking of hospital affiliations with peer review results [10]. Other studies have found alternative domain-specific objective measures to corroborate online review sites with relevant tangible outcomes, like restaurant ratings with patron visits [11].

## Results

This study examined 223,715 reviews of 41,104 unique (nonduplicated) physicians from 10 of the largest cities in the United States (Atlanta, Boston, Chicago, Dallas, Washington DC, Los Angeles, Miami, New York, Philadelphia, and San Francisco). Reviews were extracted in January 2017. Of these physicians, 1142 were included as “America’s Top Doctors” in the Castle Connolly Medical rankings. The number of ratings and physicians evaluated makes this study the largest-scale evaluation of its kind, to date. The profile of the overall sample is provided in Table 1. Specific elements extracted included doctor name, rating (numeric), number of reviews, specialization, source (ratings site), city, and state. To mitigate issues related to “fake” reviews as well as influential observations, we excluded any physician with fewer than three reviews and specializations with fewer than five reviews. Of the total number of physicians with reviews, 16,525 had fewer than three reviews, making the final analyzed sample size of physicians 24,579 (Multimedia Appendix 1).

From Multimedia Appendix 1, four specializations demonstrated differences in online patient average ratings between those physicians included in Castle Connolly Medical’s listing of “America’s Top Doctors” and those not listed: allergists, family medicine, internists, and pediatricians. For each of these specializations, those physicians with a listing in Castle Connolly Medical received a higher rating than those physicians not listed. The remaining specializations exhibited little difference between physicians listed and those not listed.

**Table 1:** Rated physicians by source.

Ratings source	Number of physicians^a^	Number of reviews	Average rating (1-5)
HealthGrades	17,385	113,427	3.97
RateMDs	19,631	72,228	3.83
Vitals	4088	38,060	4.06
Total	41,104	223,715	3.91

^a^Nonduplicated, unique number of physicians.

## Discussion

### Principal Findings

This study sought to determine the validity of patient ratings for physicians by evaluating the mean online ratings for physicians, by specialty, between those who had been nominated by their peers as one of “America’s Top Doctors” or not, as reported by Castle Connolly Medical. We found that four specializations demonstrated differences in ratings between those physicians included in Castle Connolly Medical’s listing of “America’s Top Doctors” and those not listed: allergists, family medicine, internists, and pediatricians. Specifically, our study found that the validity of patient online reviews of physicians varies by specialization. This finding has implications related to how patients make choices related to health care.

Physicians have been inundated with mandates for attaining the “triple aim” of reducing costs and increasing patient experiences and quality [12]. In doing so, many have moved to a model of “patient-centered care” which seeks to form continuous patient-physician relationships [13]. Thus, some practices have simultaneously begun to direct attention at both the nature of the relationship and the quality of that encounter. Given that these efforts appear to be primarily directed at more “primary care” and “in-office” settings, our finding that patient reviews are valid for specializations that could be characterized as primarily “in-office” settings is not unexpected.

Within the context of promoting competition, information transparency needs to be both complete and understood. This review would suggest that online patient ratings accomplish neither of these market objectives. In fact, there may be implications for shopping behavior to negatively influence quality of care outcomes; care continuity is associated with many positive health outcomes including decreased hospitalizations, fewer emergency room visits, lower health care costs, and improvements in the use of preventative care services [14]. Conversely, evidence indicates that patients who experience more fragmented primary care services also have patterns of care that more significantly deviate from determined best practice guidelines and result in higher overall health care costs. Negative reviews could thus promote “doctor shopping” based on incomplete or nonfactual information and lead to more fragmented care continuity, and potentially less optimal health outcomes [15,16].

Health systems have called for more holistic approaches to treating patients and placing measurable value on attributes such as trust and continuity of care [17]. In a recent edition of the Journal of the American Medical Association, physicians discussed the role that standardized quality assessment tools have on care practice and the need to be thoughtful when constructing such measures [18]. Physician rating websites have utility, but are imperfect proxies for competence [19,20]. If such questions have arisen about standard best practice measurement, even greater questions exist about unstandardized and undefined open assessments such as online patient reviews, particularly in specialties where the patient has limited direct experience with their health care provider (eg, Anesthesiology).

### Limitations

The selected basis for physician peer review for this study–Castle Connolly Medical–is not immune to challenge; while the organization does not receive payments or petitions, physicians have publicly questioned the “lobbying” efforts that some colleagues undertake to be included in their lists. However, no objective truth in the determination of a “good” or “bad” physician has been established. Other studies have explored alternative assessments for physician performance (eg, clinical outcomes, costs to treat, board certifications) and have acknowledged a variety of issues and limitations related to associating reviews with performance [21,22].

The current study only incorporated average numerical results for physicians (rather than an individual numeric rating for each review) from the three ratings sources; text from reviews was not analyzed. While the patterns and general findings would likely not change based upon text analysis, the text may provide additional insights regarding frequently occurring terms or relevant patterns for interested researchers.

We were not able to ascertain details about the individuals providing the ratings. Specifically, this study did not consider the patients’ insurance type. This insurance type could affect how a patient experiences the service provided relative to perceived value; those with higher out-of-pocket direct costs via copays and/or high deductibles may be more cost sensitive and therefore more likely to “shop” for health care in the face these payments.

### Conclusions

A deceptive review or set of reviews related to a hotel visit is an inconvenience, but decisions based on deceptive or poorly-informed patient reviews related to a health care provider could have dire consequences for an individual using these reviews to inform their health care-related decisions. The presence of online ratings sites will likely continue to grow and expand across all segments of the economy. The results of this large-scale study indicate that while patient ratings are consistent with physician peer review ratings for specialties like allergists and pediatricians, patient reviews were not consistent with medical peer review for specializations characterized by less patient contact (eg, anesthesiology). This result may indicate that patients are not sufficiently knowledgeable to provide informed physician ratings for some medical specializations, leading other information-seekers to potentially less-qualified providers.

## Appendix

Multimedia Appendix 1 Overall mean ratings by specialization for physicians listed and not listed in Castle Connolly Medical.
