# Supplementary material for: The Validity of Online Patient Ratings of Physicians: Analysis of Physician Peer Reviews and Patient Ratings
Source: Interact J Med Res. 2018 Apr 9;7(1):e8. doi: 10.2196/ijmr.9350 (PMC5913572; doi:10.2196/ijmr.9350)
Supplement: Multimedia Appendix 1 [file ijmr_v7i1e8_app1.pdf]

Multimedia Appendix 1. Overall Mean Ratings by Specialization for Physicians listed and not listed ir

| | Overall | | | Listed in Castle Connolly | |
|---|---|---|---|---|---|
| | Number of Physicians[a] | Mean Reviews per Physician | Rating Mean (SD) | Number of Physicians | Mean Reviews per Physician |
| Overall | 24,579 | 9.1 | 3.85 (0.90) | 622 | 8.57 |
| | | | | | |
| Site | | | | | |
| Healthgrades.com | 11,954 | 9.44 | 3.89 (0.91) | 111 | 9.62 |
| RateMDs.com | 9,672 | 7.38 | 3.74 (0.91) | 464 | 7.74 |
| Vitals.com | 2,953 | 13.38 | 4.09 (0.76) | 47 | 14.28 |
| | | | | | |
| Type of Specialty | | | | | |
| Allergists / Immunologists | 743 | 7.82 | 3.91 (0.86) | 13 | 6.15 |
| Cardiologists | 1,079 | 6.73 | 4.21 (0.77) | 35 | 5.46 |
| Dermatologists | 1,692 | 10.47 | 3.76 (0.89) | 33 | 11.33 |
| Endocrinologists | 1,273 | 12.17 | 3.64 (0.87) | 35 | 9.11 |
| ENT Doctors | 310 | 7.42 | 3.75 (0.89) | 22 | 6.68 |
| Family Medicine | 3,003 | 7.55 | 3.73 (0.92) | 61 | 7.26 |
| Gastroenterologists | 1,577 | 9.47 | 3.79 (0.88) | 52 | 8.21 |
| General Surgeons | 514 | 9.35 | 4.03 (0.83) | 30 | 8.97 |
| Gynecologists | 1,164 | 10.21 | 3.68 (0.85) | 37 | 8.16 |
| Hematology and Oncology | 323 | 7.61 | 3.94 (0.87) | 23 | 6.7 |
| Internal Medicine | 1,680 | 8.62 | 3.92 (0.93) | 10 | 8.4 |
| Neurologists | 795 | 9.28 | 3.85 (0.82) | 56 | 11.14 |
| Obstetrics and Gynecology | 1,751 | 11.34 | 3.89 (0.88) | 17 | 9.65 |
| Ophthalmology | 1,238 | 9.13 | 4.01 (0.84) | 15 | 12.67 |
| Orthopedic Surgeons | 1,796 | 12.65 | 3.89 (0.86) | 68 | 8.49 |
| Pediatricians | 886 | 6.36 | 3.91 (0.92) | 26 | 7.31 |
| Pulmonologists | 124 | 6.65 | 3.71 (0.90) | 9 | 4.22 |
| Rheumatologists | 153 | 9.69 | 3.77 (0.82) | 15 | 11.73 |
| Urology | 286 | 8.66 | 3.77 (0.83) | 21 | 9.95 |

Notes:
a. Only physicians with 3 or more ratings were included and only specialities with more than 5 phys
b. Based on Mann Whitney test

ı Castle Connolly Medical.

| Rating Mean (SD) | Not Listed in Castle Connolly<br>Number of Physicians | Mean Reviews per Physician | Rating Mean (SD) | Difference in Mean Rating For Listed versus Not Listed (95% CI) | p-value[b] |
|---|---|---|---|---|---|
| 3.94 (0.80) | 23,957 | 9.12 | 3.85 (0.90) | 0.09 (0.15,0.3) | 0.0793 |
| | | | | | |
| 4.06 (0.72) | 11,843 | 9.44 | 3.89 (0.91) | 0.17 (0.30,0.03) | 0.1043 |
| 3.88 (0.83) | 9,208 | 7.36 | 3.73 (0.91) | 0.15 (0.23,0.07) | 0.0014 |
| 4.27 (0.61) | 2,906 | 13.37 | 4.08 (0.76) | 0.18 (0.40,-0.03) | 0.1232 |
| | | | | | |
| 4.49 (0.39) | 730 | 7.85 | 3.90 (0.86) | 0.60 (0.84,0.36) | 0.0146 |
| 4.02 (0.68) | 1,044 | 6.77 | 4.21 (0.78) | -0.19 (0.07,-0.45) | 0.0623 |
| 3.67 (0.79) | 1,659 | 10.45 | 3.76 (0.89) | -0.09 (0.21,-0.40) | 0.3701 |
| 3.70 (0.84) | 1,238 | 12.26 | 3.64 (0.87) | 0.06 (0.35,-0.23) | 0.7338 |
| 3.79 (0.93) | 288 | 7.48 | 3.75 (0.89) | 0.04 (0.43,-0.35) | 0.785 |
| 4.38 (0.51) | 2,942 | 7.55 | 3.71 (0.92) | 0.67 (0.80,0.53) | <.0001 |
| 3.96 (0.80) | 1,525 | 9.51 | 3.79 (0.88) | 0.17 (0.42,-0.07) | 0.1854 |
| 4.05 (0.63) | 484 | 9.37 | 4.03 (0.84) | 0.03 (0.33,-0.28) | 0.6327 |
| 3.53 (0.88) | 1,127 | 10.28 | 3.69 (0.85) | -0.15 (0.13,-0.43) | 0.2905 |
| 4.02 (1.06) | 300 | 7.68 | 3.94 (0.86) | 0.08 (0.45,-0.29) | 0.3366 |
| 4.42 (0.30) | 1,670 | 8.62 | 3.92 (0.94) | 0.50 (0.72,0.28) | 0.0525 |
| 3.94 (0.69) | 739 | 9.14 | 3.84 (0.83) | 0.10 (0.29,-0.09) | 0.5561 |
| 3.85 (0.87) | 1,734 | 11.36 | 3.89 (0.88) | -0.05 (0.38,0.47) | 0.8403 |
| 4.07 (0.44) | 1,223 | 9.09 | 4.01 (0.84) | 0.06 (0.31,-0.19) | 0.7812 |
| 3.81 (0.80) | 1,728 | 12.81 | 3.89 (0.87) | -0.08 (0.13,-0.29) | 0.2656 |
| 4.58 (0.64) | 860 | 6.33 | 3.89 (0.92) | 0.70 (0.96,0.43) | <.0001 |
| 3.75 (1.01) | 115 | 6.84 | 3.71 (0.90) | 0.04(0.66,-0.58) | 0.9385 |
| 3.55 (0.48) | 138 | 9.47 | 3.79 (0.85) | -0.24 (0.05,-0.54) | 0.1 |
| 3.87 (0.73) | 265 | 8.56 | 3.76 (0.84) | 0.11 (0.48,-0.26) | 0.6072 |

icians were included
